# A Portable Neck-Surface Piezoelectric Sensor for Evaluating Subclinical Carotid Atherosclerosis via Snoring Vibratory Analysis: An Exploratory Dual-Modality Study

**DOI:** 10.3390/bios16080428

**Published:** 2026-08-06

**Authors:** Li-Ang Lee, Li-Pang Chuang, Guo-She Lee, Cheng-Kuo Lai, Huei-Dan Cheng, Zi-Xuan Huang, Zong-Han Lee, Liang-Yu Shyu, Hsueh-Yu Li, Chi-Hung Liu, Yi-Ping Chao

**Affiliations:** 1 Department of Otorhinolaryngology-Head and Neck Surgery, Sleep Center, Linkou Chang Gung Memorial Hospital, Taoyuan City 33305, Taiwan; 5738@cgmh.org.tw (L.-A.L.); hyli38@cgmh.org.tw (H.-Y.L.); 2 School of Medicine, College of Life Science and Medicine, National Tsing Hua University, Hsinchu City 300044, Taiwan; 3 School of Medicine, College of Medicine, Chang Gung University, Taoyuan City 33302, Taiwan; r5243@cgmh.org.tw (L.-P.C.); ivanliu001@cgmh.org.tw (C.-H.L.); 4 Department of Pulmonary and Critical Care Medicine, Sleep Center, Linkou Chang Gung Memorial Hospital, Taoyuan City 33305, Taiwan; 5 Faculty of Medicine, National Yang Ming Chiao Tung University, Taipei City 112304, Taiwan; kslee3@vghtpe.gov.tw; 6 Department of Otolaryngology, Taipei Veterans General Hospital, Taipei City 112201, Taiwan; 7 Largan Health AI-Tech Co., Ltd., Taipei City 114066, Taiwan; chris.lai@largan-health.com (C.-K.L.); denise.cheng@largan-health.com (H.-D.C.); 8 Department of Biomedical Engineering, Chung Yuan Christian University, Taoyuan City 320314, Taiwan; hi678925@gmail.com (Z.-X.H.); h85260505@gmail.com (Z.-H.L.); liangyu2@cycu.edu.tw (L.-Y.S.); 9 Departments of Neurology, Linkou Chang Gung Memorial Hospital, Taoyuan City 33305, Taiwan; 10 Department of Computer Science and Information Engineering, Chang Gung University, Taoyuan City 33302, Taiwan

**Keywords:** obstructive sleep apnea syndrome, snoring sound energy, snoring vibration energy, carotid artery profile, ambient microphone, neck-surface piezoelectric sensor, hierarchical multivariable regression

## Abstract

Obstructive sleep apnea syndrome (OSAS) is heavily implicated in subclinical cardiovascular disease; however, traditional polysomnographic metrics fail to capture the localized mechanical trauma exerted on the carotid artery. To address this methodological gap, this study evaluated exploratory associations between frequency-domain snoring characteristics and right carotid artery alterations in 50 patients with OSAS. Snoring was quantified using a dual-modality bioelectronic approach: an ambient microphone captured airborne snoring sound energy (SSE), while a portable neck-surface piezoelectric sensor recorded tissue-conducted snoring vibratory energy (SVE). Subclinical vascular changes, including carotid intima-media thickness (CIMT) and atherosclerosis, were assessed via ultrasonography. Hierarchical multivariable regression demonstrated that acoustic SSE%-404–500 Hz and mechanical SVE%-112–144 Hz independently correlated with preliminary CIMT increases (adjusted *β* = 0.033 and 0.021, respectively; both *p* < 0.05), alongside neck circumference. Conversely, SSE%-404–500 Hz emerged as an exploratory marker for focal carotid atherosclerosis (adjusted odds ratio = 1.828; *p* = 0.009). Integrating this metric with baseline parameters yielded exploratory diagnostic capacity (area under the curve = 0.833; *p* < 0.001), achieving 89% sensitivity and 69% specificity. These findings suggest that dual-modality spectral analysis provides a non-invasive exploratory framework for cardiovascular risk stratification, isolating localized mechanotransduction phenotypes independently of systemic hypoxia.

## 1. Introduction

Stroke and atherosclerotic cardiovascular disease (CVD) are frequently driven by subclinical structural changes within the carotid artery [1,2]. While traditional risk factors are well-documented, the biomechanical trauma generated by sleep-disordered breathing (SDB) is increasingly recognized as a potent, modifiable catalyst for vascular remodeling [3,4]. Habitual snoring, observed in over half of some middle-aged populations [4,5], is no longer viewed merely as an acoustic nuisance [6]. Instead, chronic, high-intensity snoring functions as a direct mechanical insult to the cervical vasculature, accelerating carotid intima-media thickness (CIMT) and high-risk plaque formation independently of severe hypoxemia [7,8].

Despite this paradigm shift, mapping the precise trajectory from airway vibration to endothelial injury remains technically challenging. Animal models strongly support a localized “response-to-injury” pathway, demonstrating that propagated snoring vibrations trigger proinflammatory responses and endothelial dysfunction in the carotid wall without requiring a hypoxic environment [9]. Translating these mechanisms to humans, however, is obstructed by inadequate assessment protocols. Epidemiological reliance on self-reported questionnaires introduces severe recall bias [10,11], while conventional polysomnography struggles to untangle the physical impact of snoring from the systemic sympathetic arousal characteristic of obstructive sleep apnea syndrome (OSAS) [12,13]. Consequently, the distinct roles of specific vibrational frequencies in human mechanotransduction [14]—as well as outcome variations driven by sex or neck adiposity [15]—remain poorly defined, leading to conflicting longitudinal evidence regarding CIMT progression [10].

To accurately quantify this dose–response relationship, the field must pivot toward advanced, objective bioelectronic measurement [15]. Standard ambient microphones are fundamentally compromised by environmental noise and air attenuation; operating as “open systems,” they capture airborne snoring sound energy (SSE) but drastically underestimate the true tissue-conducted snoring vibratory energy (SVE) assaulting the carotid artery [5,16,17]. To bridge this technological gap, we developed a portable, neck-surface piezoelectric sensor (NPS) [17,18]. Functioning as a closed-system bioelectronic device, the NPS directly acquires SVE and local hemodynamic data without external acoustic contamination [17]. While our previous computational studies (including deep learning algorithms) successfully utilized this system to distinguish severe sleep apnea from primary snoring [18], the capability of targeted SVE to stratify specific carotid profiles—ranging from normal to thickened CIMT and focal atherosclerosis—remains unexplored.

Therefore, this study investigates the exploratory associations between high-resolution snoring energy spectra and subclinical vascular changes. Specifically, we aim to (1) validate the diagnostic fidelity of the NPS against traditional ambient microphones, and (2) determine whether tissue-conducted SVE offers superior exploratory predictive capacity compared to airborne SSE in evaluating right carotid artery profiles.

## 2. Materials and Methods

### 2.1. Ethical Approval and Study Oversight

The Institutional Review Board of the Chang Gung Medical Foundation (Taoyuan, Taiwan) authorized the protocol for this prospective cross-sectional analysis (No. 201700902A3) [7]. We established and maintained all clinical procedures in rigorous alignment with the 1964 Declaration of Helsinki and its modern amendments [19], while strictly observing established publication ethics for medical research [20]. Prior to any clinical assessment or data collection, the research team thoroughly briefed every participant on the scientific aims, methodological scope, and privacy safeguards of the project, successfully securing their voluntary, written informed consent.

### 2.2. Participant Recruitment and Selection Criteria

Between May 2018 and March 2020, we assembled a prospective cohort by consecutively enrolling adults presenting to our tertiary care facility for the surgical management of SDB. To cleanly isolate the exploratory vascular phenotypes under investigation, we established strict enrollment boundaries designed to suppress external confounders. Eligible candidates were restricted to those aged 20 to 59 years, possessing a body mass index (BMI) between 18.0 and 35.0 kg/m^2^, and demonstrating the capacity to provide full informed consent.

We rigorously excluded individuals whose pre-existing health profiles might obscure the evaluation of mechanically associated vascular changes. Consequently, we disqualified anyone with a history of upper airway interventions (such as uvulopalatopharyngoplasty), severe psychiatric conditions, or active cardiovascular disease. Most crucially, to ensure that established systemic risk factors did not skew our baseline structural assessments, we rejected any patient with a known history of carotid artery pathology [7]. This mandated the exclusion of anyone with previously diagnosed atherosclerosis, documented plaque formation, or known intimal-medial thickening prior to the study [7].

### 2.3. Clinical and Demographic Assessment

To establish a robust baseline profile, we systematically cataloged each participant’s anthropometric and physiological markers upon admission. This baseline phenotyping captured standard metrics—including age, biological sex, BMI, and neck circumference (NC)—alongside systolic and diastolic blood pressure (SBP and DBP) readings. We concurrently screened for classical drivers of atherosclerotic cardiovascular disease [7,21]. Within this framework, elevated cardiovascular risk was defined by the presence of advanced age (males ≥ 45 years; females ≥ 55 years), elevated body mass (BMI ≥ 24 kg/m^2^), active or historical tobacco use, clinical hypertension, diabetes mellitus, or hyperlipidemia [7,21].

Beyond objective biometrics, we quantified subjective sleep pathology and the broader disease burden using two standardized self-reporting frameworks:

#### 2.3.1. Epworth Sleepiness Scale (ESS)

We deployed this 8-item inventory to gauge waking somnolence, classifying aggregate scores above 10 as clinical manifestations of excessive daytime sleepiness [22].

#### 2.3.2. Snore Outcomes Survey (SOS)

To capture the psychosocial and physical footprint of sleep-disordered breathing, we utilized the SOS. This instrument provided a composite evaluation of the patients’ snoring severity—specifically highlighting loudness and chronicity—and its disruptive impact on their daily quality of life [23].

### 2.4. Nocturnal Polysomnography and Respiratory Event Adjudication

To establish a gold-standard baseline for sleep architecture and respiratory mechanics, every subject completed a continuous, technician-supervised overnight polysomnography evaluation utilizing the Alice 5 diagnostic platform (Philips Respironics, Murrysville, PA, USA). Because pristine signal acquisition was paramount for our concurrent acoustic and vibratory sensor testing, we isolated all overnight recordings within a specialized, acoustically shielded sleep laboratory to block external noise contamination [24].

The physiological monitoring array captured sleep staging and cardiac rhythms via continuous multi-channel electroencephalography, electrooculography, electrocardiography, and targeted electromyography (tibial and submental).

Credentialed sleep specialists manually reviewed and scored all respiratory disturbances, strictly adhering to the 2017 American Academy of Sleep Medicine standardized protocols [25,26]. Under these criteria, apneas were flagged when peak thermal sensor amplitude collapsed by 90% or more for at least 10 s. Hypopneas were defined by a minimum 30% drop in nasal pressure excursions lasting 10 s or longer, obligatorily coupled with either a transient cortical arousal or a 4% reduction in baseline oxyhemoglobin saturation [27].

Finally, we quantified global disease severity by calculating the apnea–hypopnea index (AHI), representing the average frequency of these combined respiratory events per hour of total sleep time. A clinical diagnosis of obstructive sleep apnea was confirmed for any participant registering an AHI of 5 or greater events per hour. To comprehensively map the concurrent systemic hypoxic burden, the diagnostic array also logged the 3% oxygen desaturation index (ODI3), alongside the mean and absolute minimum pulse oximetry (SpO_2_) readings.

### 2.5. Ultrasonographic Evaluation of the Common Carotid Artery

To preserve diagnostic neutrality, the ultrasound technicians who captured and analyzed the CIMT measurements operated under strict blinding protocols. Specifically, they were completely blinded to the patients’ polysomnographic results (including AHI and severity grouping), to all acoustic/vibratory spectral data, physical measurements, and medical backgrounds [7].

**Figure 1:**
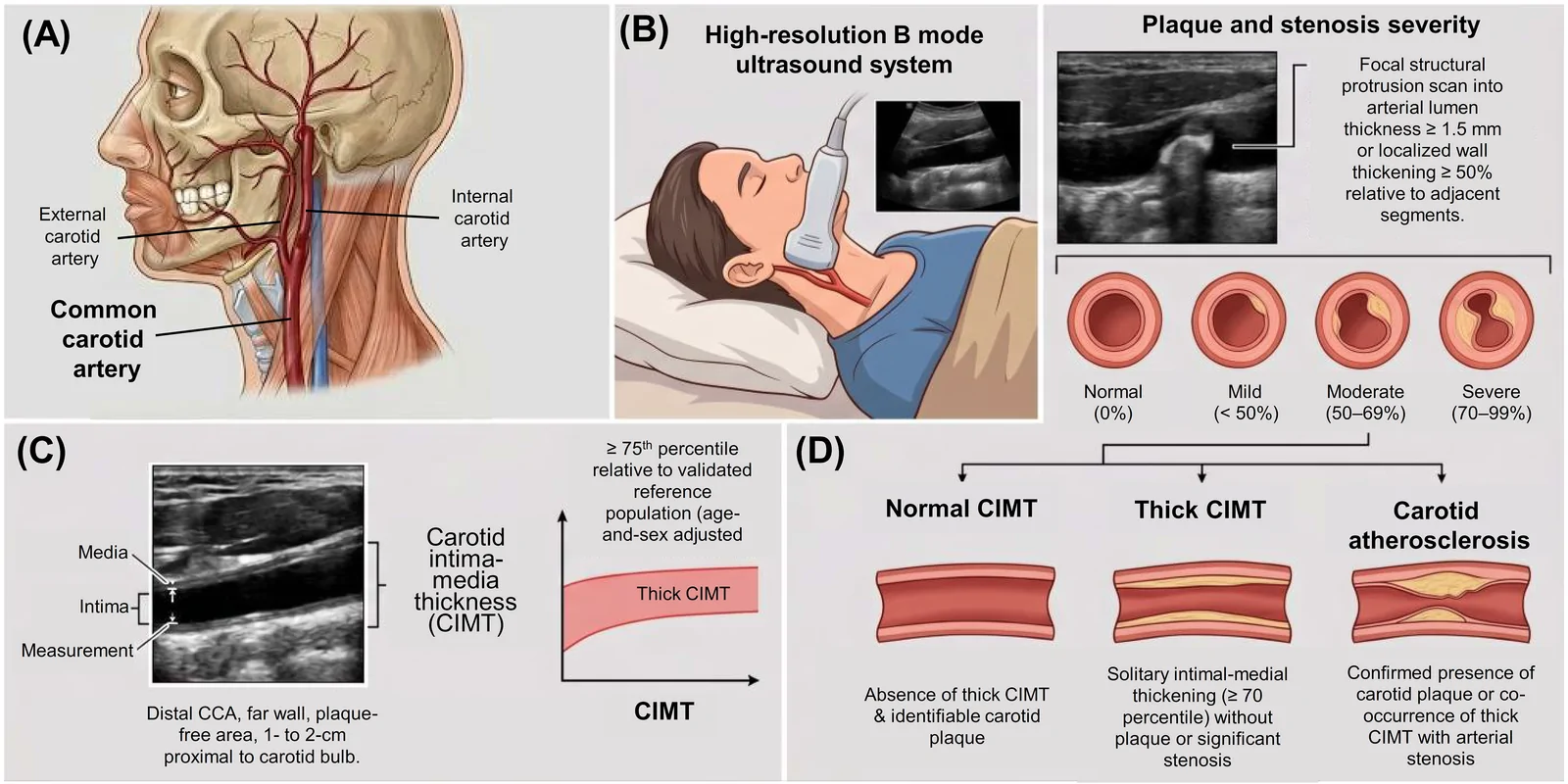
Clinical workflow for defining exploratory carotid artery phenotypes via targeted ultrasonography. (**A**) Schematic overview mapping the common, internal, and external carotid branches. (**B**) Procedural setup illustrating real-time B-mode sonography. A 5–13 MHz linear array transducer captures high-resolution cervical vascular imaging while the subject rests in a supine position. (**C**) Quantification of carotid intima-media thickness (CIMT). Sonographers target a clear, plaque-free region spanning 1 to 2 cm just upstream from the carotid bulb on the distal far wall. A “Thick CIMT” designation requires the averaged structural depth to reach or exceed the 75th percentile of an age- and sex-matched normative demographic. (**D**) Diagnostic thresholds for atherosclerotic plaque and luminal narrowing. A discrete lesion protruding inward by at least 1.5 mm, or regional tissue expansion exceeding 50% of the surrounding healthy wall, constitutes a confirmed plaque. Concurrently, arterial stenosis is graded by luminal diameter loss: normal (0%), mild (< 50%), moderate (50–69%), or severe (70–99%). Final triage of subjects into three progressive vascular phenotypes: Normal CIMT (structurally intact without thickening or lesions), Thick CIMT (isolated dimensional thickening devoid of plaques or narrowing), and Carotid Atherosclerosis (definitive plaque visualization or the pathological pairing of thickened CIMT with measurable stenosis).

Subclinical arterial damage was quantified primarily through the carotid intima-media thickness (CIMT) along the far wall of the right distal common carotid artery (CCA) (Figure 1A). While patients rested in a supine posture, technicians utilized a 5–13 MHz linear array transducer paired with a high-resolution B-mode ultrasound (Philips HDI 5000 System, ATL-Philips, Bothell, WA, USA) to image the cervical vasculature (Figure 1B). During these scans, technicians deliberately targeted clear, plaque-free zones located exactly 1 to 2 cm upstream from the carotid bulb [26].

We extracted six distinct thickness readings from the right CCA and calculated their mean to ensure analytical stability (Figure 1C). Following the American Society of Echocardiography guidelines, we classified a patient as having “thick CIMT” if their averaged measurement hit or exceeded the 75th percentile of a demographically matched, age- and sex-adjusted reference cohort [21,27].

The presence of carotid plaque required specific visual confirmation: either a discrete luminal intrusion measuring at least 1.5 mm in depth, or a regional wall expansion exceeding 50% of the thickness of neighboring healthy tissue [28,29] (Figure 1D). Sonographers further graded arterial stenosis by calculating the luminal diameter reduction, classifying the narrowing as normal (0%), mild (< 50%), moderate (50–69%), or severe (70–99%) [28,29].

By synthesizing these anatomical markers—intimal-medial thickening, focal plaque, and luminal stenosis—we triaged the subjects into three progressive vascular phenotypes to serve as our exploratory baseline targets (Figure 1D):

1. Normal CIMT: A completely unremarkable structural profile lacking both intimal thickening and identifiable plaque.
2. Thick CIMT: An isolated elevation in intimal-medial dimensions (≥75th percentile) without any concurrent stenotic narrowing or plaque deposition.
3. Carotid Atherosclerosis: The definitive structural presence of carotid plaque, or a pathological combination of abnormal CIMT coupled with measurable arterial stenosis.

### 2.6. Acoustic Snoring Sound Acquisition and Spectral Analysis

**Figure 2:**
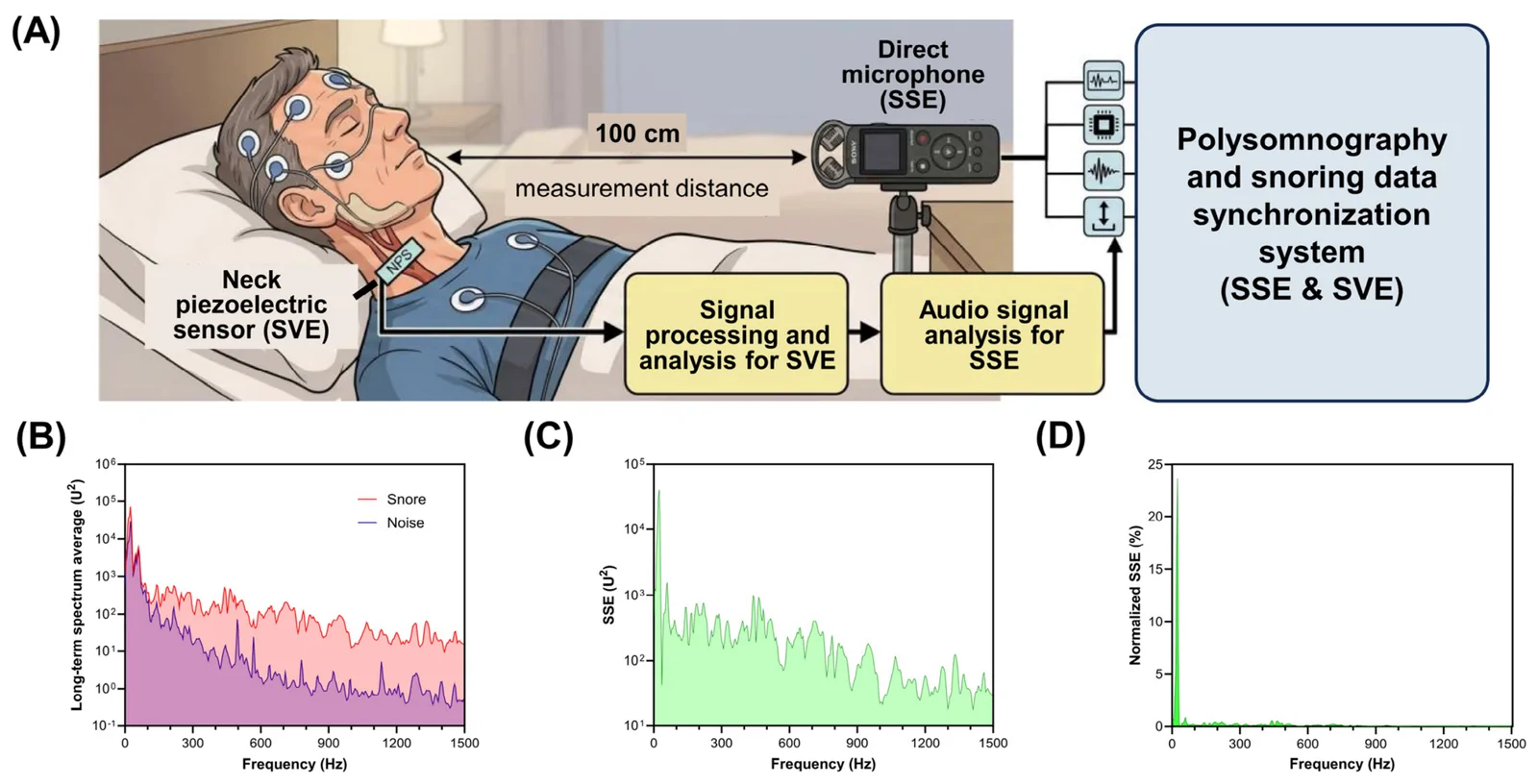
Dual-modality hardware configuration and acoustic spectral analysis workflow. (**A**) Schematic of the parallel data capture architecture. A customized portable neck-surface piezoelectric sensor anchors the closed-system acquisition of snoring vibratory energy (SVE), while an overhead directional microphone (fixed at 100 cm) records airborne snoring sound energy (SSE). Both the vibratory and audio streams are precisely time-locked to the baseline nocturnal polysomnography. (**B**) Fast Fourier transform visualization. The graph plots the raw spectral magnitude of a discrete snoring burst against the long-term spectrum average (LTSA), establishing the baseline ambient room noise across a 4–1500 Hz bandwidth. (**C**) Extraction of the net snore power. By systematically subtracting the background LTSA noise floor from the raw spectral data, the pure physiological acoustic emission of the airway obstruction is isolated. (**D**) Derivation of the normalized SSE (SSE%). To mitigate individual discrepancies in raw vocal volume, the energy at each distinct frequency is calculated and plotted as a relative percentage of the patient’s total acoustic output.

To establish a conventional airborne acoustic baseline alongside polysomnography [7,17], we positioned a high-fidelity digital audio recorder (PCM-D50, Sony Electronics Inc., Tokyo, Japan) exactly 100 cm above the participant (Figure 2A) [7,14]. This fixed spatial geometry stabilized the signal-to-noise ratio across the cohort. Audio streams were digitized at a 44.1 kHz sampling rate (16-bit resolution) and routed directly into a Labview analytical environment (Sound & Vibration Toolkit, National Instruments Corp., Austin, TX, USA).

Spectral decomposition of the raw audio was executed via fast Fourier transform, yielding a 4–1500 Hz power spectrum evaluated at 4 Hz intervals [14]. For automated event isolation, we applied Snore Master^®^ (Largan Health AI-Tech Co., Ltd., Taipei, Taiwan), a proprietary detection algorithm extensively validated in our prior work [7,14,17,30,31]. This platform screens the continuous acoustic signal in 0.25 s epochs. It registers a discrete snoring event (SE) exclusively when the signal’s spectral power breaks the ambient room noise floor by a minimum of 6 dB.

To extract the genuine physiological sound, we subtracted the room’s long-term spectrum average (LTSA) noise from the raw spectral data, defining the net snore power [14] (Figure 2B). This measurement provided the open-system comparative standard necessary to evaluate our experimental tissue-coupled sensor [17,18].

Because absolute snoring volume varies drastically due to individual anatomical differences (Figure 2C), we computed the normalized frequency-domain SSE (SSE%). By expressing the energy at any discrete frequency as a percentage of the total acoustic output [7] (Figure 2D), the SSE% standardizes the data, isolating the proportional acoustic footprint of the upper airway obstruction.

### 2.7. Snoring Vibration Acquisition and Spectral Analysis

**Figure 3:**
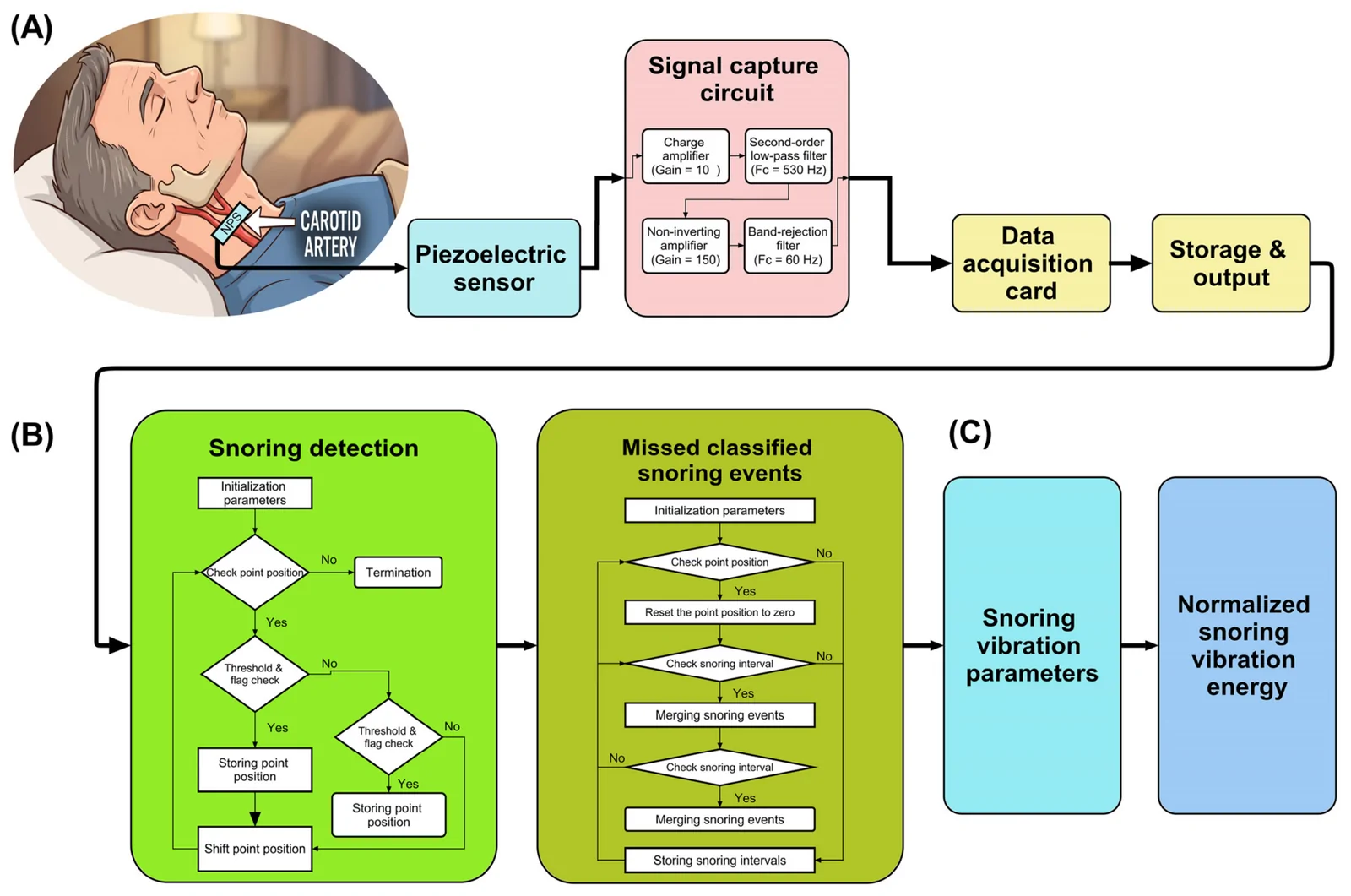
Engineering architecture and computational pipeline of the portable neck-surface piezoelectric sensor (NPS). (**A**) Hardware schematic detailing the closed-system bioelectronic device. The device captures mechanical tissue vibrations directly from the skin overlying the carotid artery using an anatomically conforming piezoelectric transducer. Prior to digitization, the analog signal is refined through a dedicated conditioning cascade, featuring a high-gain charge amplifier, a non-inverting amplifier, a second-order low-pass anti-aliasing filter (Fc = 530 Hz), and a 60 Hz band-rejection filter to eliminate electrical grid interference. (**B**) Automated algorithmic detection of snoring events. The flowchart illustrates the multi-stage signal processing logic, which decomposes the waveform via discrete wavelet transform, applies adaptive dynamic thresholding, and utilizes an error-correction protocol to screen out motion artifacts while seamlessly merging fragmented signal bursts. (**C**) Spectral analysis and energy normalization workflow. This section depicts the mathematical derivation of the normalized snoring vibration energy (SVE%), calculated by quantifying the mechanical power within targeted frequency sub-bands as a relative percentage of the entire broadband spectrum.

To capture the true mechanical stress applied to the cervical vasculature, we utilized our custom-engineered portable NPS [17,18]. Operating strictly as a closed system, the NPS avoids the acoustic interference inherent to open-air microphones, thereby preserving the genuine mechanotransduction profile of the snoring vibrations. We secured the device over the right carotid triangle, positioning it immediately adjacent to the common carotid artery (Figure 3A).

#### 2.7.1. Hardware Architecture of the NPS System

The physical sensor platform integrates three primary modules:

1. Piezoelectric Transducer: The sensing module employs a lead zirconate titanate ceramic (PZT) element (40 × 10 × 0.5 mm; Eleceram Technology Co., Ltd., Taoyuan, Taiwan) fabricated from Ka-type ceramic. This material provides high piezoelectric sensitivity (d_33_ = 470 pC/N), low dielectric loss (tan δ = 1.5%), and a stable elastic modulus, enabling a linear, low-hysteresis response across the physiological vibration band (0.01–530 Hz). The sensor operates well below its structural resonance (>1 kHz), maintaining flat sensitivity with nonlinearity <1% and hysteresis <0.5%. Signal acquisition was performed at ≥10 kHz to ensure adequate temporal resolution. To guarantee stable coupling and user comfort, the PZT element was housed within an ergonomically contoured, 3D-printed plastic casing (51 × 21 × 14 mm) shaped to the cervical anatomy, with a light, uniform preload (0.5–2 N) that yielded repositioning repeatability within <3%.
2. Signal Conditioning Circuitry: The raw piezoelectric voltage undergoes rigorous preprocessing to resolve micro-deformations. The conditioning circuit utilizes a high-gain charge amplifier (OPA2333, Texas Instruments, Dallas, TX, USA; gain = 10^8^), followed by a non-inverting amplifier (gain = 150) and a second-order low-pass anti-aliasing filter (Fc = 530 Hz). A dedicated 60 Hz band-rejection filter neutralizes power-line artifacts to ensure spectral purity [32].
3. Digitization Interface: Conditioned analog outputs are digitized using a 12-bit data acquisition module (USB-6008 DAQ, National Instruments Corp., Austin, TX, USA). This architecture securely captures physiological frequencies ranging from 0.01 to 530 Hz at an elevated resolution.

#### 2.7.2. Computational SVE Analytical Pipeline

We built a custom computational signal-processing environment in MATLAB (version R2014a, MathWorks Corp., Natick, MA, USA) to interpret the continuous mechanical data.

1. Signal Decomposition: Complex vibratory waveforms are separated into distinct biological components via a discrete wavelet transform (DWT) utilizing a fourth-order Daubechies (db4) basis function [33,34]. By extracting Level 1 (high-frequency transients) and Level 3 (primary snoring frequencies), the algorithm isolates the critical dynamic range of the tissue vibrations.
2. Automated Event Detection: SEs are algorithmically isolated through a cascading logic sequence (Figure 3B). First, the DWT-reconstructed signals are squared to magnify burst energy, then smoothed via a 130-point moving average filter [35]. Next, adaptive dynamic thresholding compensates for patient-specific variability in snoring intensity [36]. Finally, a temporal error-correction protocol scrubs non-respiratory motion artifacts and seamlessly merges fragmented bursts. This specific NPS logic yields an 83% baseline accuracy against acoustic standards [17], which scales to 93% when paired with our advanced recurrent convolutional neural networks [18].

#### 2.7.3. Spectral Analysis and SVE Normalization

To accurately quantify the mechanical energy, the raw time-series data is passed through a third-order finite impulse response filter spanning 0.01 to 504 Hz (Figure 3C). The algorithm segments the extracted SEs into 10 s analysis windows. A fast Fourier transform (FFT) was performed on each window (frequency range 0.01–504 Hz; resolution 36 Hz) to generate a frequency power spectrum. This specific 36 Hz resolution resulted from the specific windowing and zero-padding parameters applied during the FFT calculation of the brief 10 s analysis epochs. The spectra were subsequently averaged to produce the LTSA [14]. Finally, to neutralize physiological variations in baseline sensor coupling, we compute the normalized SVE (SVE%). This metric evaluates the mechanical power within discrete frequency sub-bands as a relative percentage of the total localized vibratory energy [7].

### 2.8. Statistical Analysis

We executed all quantitative evaluations using SPSS (version 30.0; IBM Corp., Armonk, NY, USA) alongside GraphPad Prism (version 11.0; GraphPad Software, Boston, MA, USA).

After screening continuous variables for normality via the Shapiro–Wilk test, we summarized normally distributed parameters as means ± standard deviations (SD), and skewed data as medians paired with interquartile ranges (IQR). Categorical profiles are presented as absolute counts and relative percentages.

To detect baseline physiological differences across the three targeted carotid phenotypes, we applied one-way analysis of variance (ANOVA) for parametric data, the Kruskal–Wallis test for non-parametric data, and χ^2^ tests for nominal variables. When applicable, post hoc pairwise comparisons were rigorously adjusted using the Bonferroni method. We quantified monotonic relationships linking the device-captured acoustic/vibratory energies to structural vascular changes using Pearson or point-biserial correlation coefficients.

Because this bioelectronic validation study is fundamentally exploratory and hypothesis-generating, we deliberately avoided global multiple-comparison penalties (such as Bonferroni correction, false positive rate control, or permutation testing for the full-spectrum feature selection) during the correlation phase. This design choice mitigates Type II errors, ensuring that critical biomechanical patterns and effect sizes are not inadvertently discarded due to rigid, isolated *p*-value thresholds [37]. However, we acknowledge this inflates the Type I error rate, meaning our findings represent exploratory associations rather than definitive predictive markers.

To map the preliminary, non-causal associations between our targeted sensor metrics and the vascular phenotypes, we engineered hierarchical linear regression algorithms for continuous CIMT and multivariable logistic regressions for categorical carotid atherosclerosis. To guarantee the mathematical stability of these exploratory models, rigorously evaluated multicollinearity using variance inflation factors (VIF) [38]. Every marker integrated into the final architecture maintained a VIF of strictly less than 5, verifying the absence of redundant variance [39]. Furthermore, well-established clinical confounders were properly retained in the adjusted model while separately testing robustness to influential observations identified via Cook’s distance [40].

We report these exploratory associations as regression coefficients (*β*) or adjusted odds ratios (aORs) coupled with 95% confidence intervals (CIs). A sensitivity analysis excluding administrative staff examined whether occupational role influenced the results.

Finally, to evaluate the sensor’s diagnostic performance, we mapped curves. We isolated the optimal diagnostic threshold for identifying atherosclerosis by calculating the Youden index (J), which maximizes the sum of sensitivity and specificity minus one [41]. The threshold for statistical significance was established at a two-tailed *p* value of <0.05 across all analyses.

## 3. Results

### 3.1. Baseline Clinical Profiles of the Sensor Validation Cohort

This bioelectronic validation study enrolled 50 participants (84% male) with a mean age of 39.7 ± 9.5 years. The cohort exhibits a severe obstructive sleep apnea phenotype, characterized by high respiratory event frequencies (median AHI: 66.0 events/h; IQR: 26.4–80.2) and pronounced nocturnal hypoxemia (median ODI3: 46.1 events/h). Consistent with typical upper airway obstruction profiles, structural adiposity was highly prevalent; 84% of subjects were classified as overweight or obese, demonstrating a mean BMI of 28.39 ± 3.91 kg/m^2^ (Table 1).

Using high-resolution sonography, we successfully partitioned this testing cohort into three progressive structural targets: normal CIMT (*n* = 17, 34%), thick CIMT (*n* = 15, 30%), and definitive carotid atherosclerosis (*n* = 18, 36%).

Crucially for isolating the variables of interest, core demographic traits, subjective symptom burdens (ESS and SOS scores), and traditional polysomnographic severity metrics (AHI and minimal SpO_2_) showed no statistically significant variance across these three distinct vascular strata (all *p* > 0.05). However, hemodynamic stress markers diverged sharply. SBP fluctuated significantly across the profiles (*p* = 0.006), peaking specifically in the thick CIMT group (137.5 ± 16.4 mmHg) when compared directly against the structurally normal patients (119.6 ± 10.7 mmHg; adjusted *p* = 0.005). Similarly, clinical hypertension tracked closely with vascular degradation (*p* = 0.013); it presented frequently within the thick CIMT (40%) and atherosclerosis (17%) groups, yet was entirely absent (0%) in the normal CIMT baseline group. Although it is noteworthy that hypertension prevalence was lower in the most severe atherosclerosis group compared to the intermediate thick CIMT group, this non-progressive decline is likely a statistical artifact driven by extreme sampling error within our very small sub-cohorts.

### 3.2. Exploratory Correlations Between CIMT and High-Resolution Snoring Spectra

**Figure 4:**
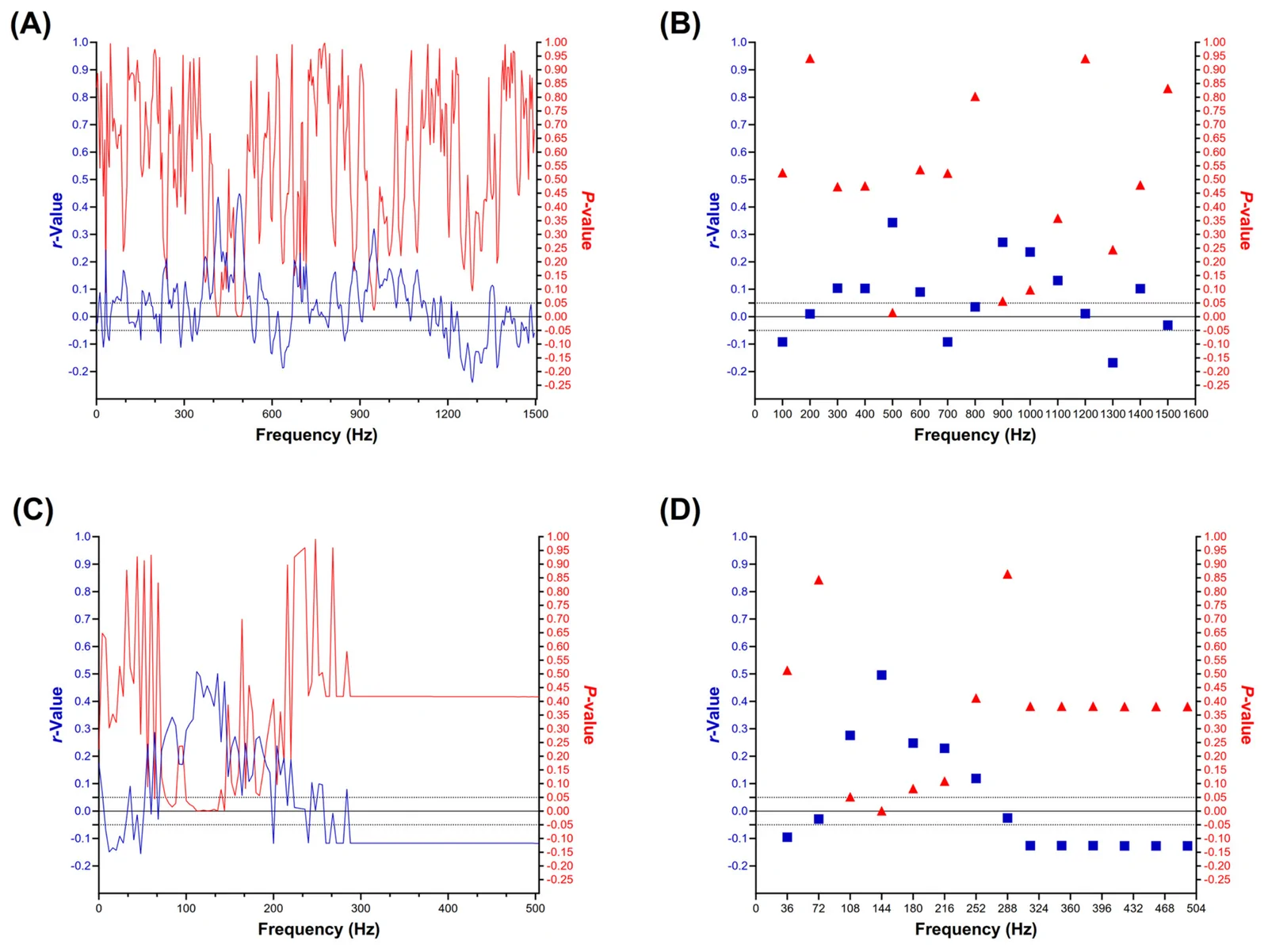
Bivariate correlation mapping between subclinical vascular phenotypes (CIMT) and high-resolution acoustic/vibratory snoring spectra. (**A**) Granular spectral analysis of airborne acoustic data. The plot traces exploratory linear associations between the depth of the CIMT and normalized snoring sound energy (SSE%), identifying distinct significance peaks at 408–420 Hz, 476–500 Hz, and 944–952 Hz. (**B**) Consolidated acoustic spectrum. This simplified visualization aggregates the airborne data, revealing a contiguous exploratory correlation band spanning 404 to 500 Hz. (**C**) Granular spectral mapping of tissue-conducted mechanics captured by the piezoelectric biosensor. The analysis isolates localized correlations between CIMT and normalized snoring vibratory energy (SVE%) at discrete mechanical frequencies: 64 Hz, 80–88 Hz, 100–136 Hz, and 144 Hz. (**D**) Consolidated mechanical spectrum. This view extracts the 112–144 Hz bandwidth as the dominant physiological association signature for intimal-medial thickening. Throughout all charts, blue plots (solid lines and squares) linked to the left vertical axis represent the Pearson correlation coefficients (r). Conversely, red plots (solid lines and triangles) linked to the right vertical axis display the corresponding *p*-values, with horizontal dashed markers defining the strict *p* = 0.05 significance boundary.

Granular spectral mapping identified discrete exploratory correlations between the acoustic data and structural vascular phenotypes. The depth of the right CIMT scaled significantly alongside SSE% localized to narrow high-frequency bandwidths: 408–420 Hz, 476–500 Hz, and 948–1500 Hz (all *p* < 0.05) (Figure 4A). Condensing these acoustic micro-bands uncovered a contiguous signature extending from 400 to 496 Hz that positively correlated with intimal-medial thickening (*r* = 0.34, *p* = 0.015) (Figure 4B).

Evaluating the tissue-conducted mechanical data, the closed-system piezoelectric sensor mapped parallel structural associations. High-resolution analysis isolated significant phenotypic links between right CIMT and SVE% clustering at discrete mechanical frequencies: 64 Hz, 80–88 Hz, 100–136 Hz, and 144 Hz (all *p* < 0.05) (Figure 4C). Consolidating this mechanical spectrum pinpointed a dominant vibratory association band spanning 112 to 144 Hz, which demonstrated a highly robust positive correlation with subclinical vascular expansion (*r* = 0.50, *p* < 0.001) (Figure 4D).

### 3.3. Spectral Energy Characteristics of the Validation Cohort

Table 2 delineates the spectral energy distributions captured by both the ambient microphone and the piezoelectric sensor. Evaluating the airborne acoustic profile, the baseline energy predominantly clustered in the infrasonic to low-frequency tier (4–100 Hz), generating an average of 66.35 ± 21.73% of the global sound output across the testing cohort. When assessing exploratory phenotypic associations, only the mid-frequency acoustic band (404–500 Hz) demonstrated a statistically significant variation across the three structural carotid groups (*p* = 0.010). Specifically, Bonferroni-adjusted post hoc testing revealed that patients harboring definitive carotid atherosclerosis generated a disproportionately high acoustic footprint within this 404–500 Hz range (3.53 ± 2.29%) compared to subjects exhibiting a structurally normal CIMT profile (1.62 ± 1.49%; adjusted *p* = 0.010). The remainder of the airborne acoustic spectrum yielded no significant categorical stratification.

Switching to the closed-system mechanical data, the piezoelectric sensor revealed that tissue-propagated vibratory energy (SVE%) was heavily weighted toward the extreme low-end spectrum. The 4–36 Hz bandwidth monopolized 93.72 ± 17.81% of the total recorded mechanical force. Interestingly, unlike the isolated mid-frequency airborne sound, evaluating the discrete mechanical energy fractions across the full 4–504 Hz vibratory spectrum did not independently yield statistically significant categorical differences among the normal CIMT, isolated thick CIMT, and advanced atherosclerosis cohorts (all *p* > 0.05).

### 3.4. Bivariate Mapping of Vascular Phenotypes and Sensor-Derived Mechanics

**Figure 5:**
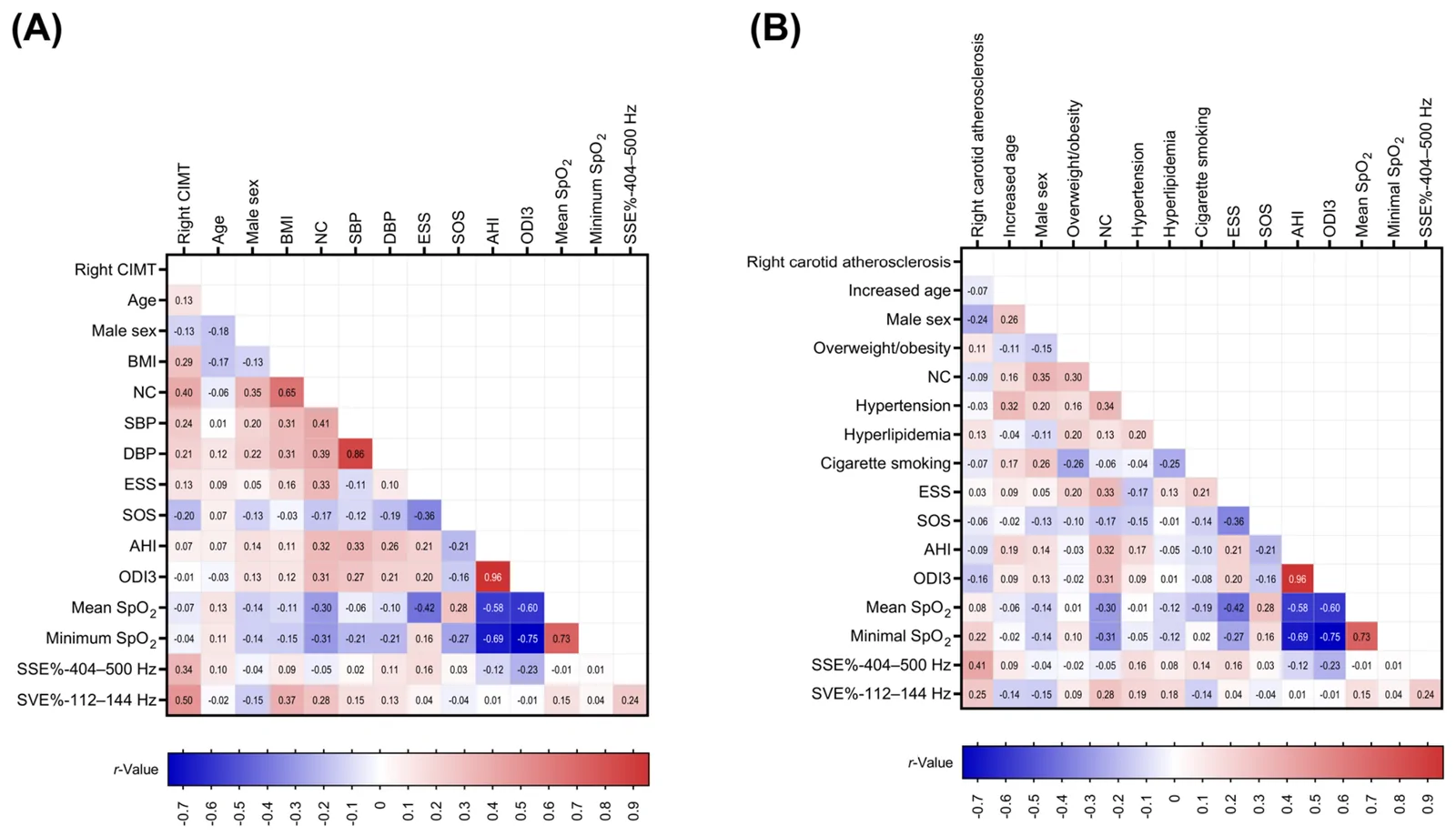
Bivariate heatmap visualizations mapping exploratory correlations between sensor-derived spectral energies, baseline clinical metrics, and structural vascular phenotypes. (**A**) Exploratory mapping of continuous carotid intima-media thickness (CIMT). This matrix highlights that progressive right CIMT thickening scales positively and significantly with physical adiposity markers (body mass index and neck circumference), alongside two distinct sensor profiles: mid-frequency airborne acoustic energy (SSE%-404–500 Hz) and localized tissue-conducted mechanical vibrations (SVE%-112–144 Hz). (**B**) Exploratory mapping of categorical atherosclerotic disease. The discrete presence of a carotid atherosclerosis profile demonstrates a positive, significant phenotypic association with elevated acoustic energy in the 404–500 Hz bandwidth. Across both visual plots, embedded cell integers denote the calculated correlation coefficients (*r*). The applied chromatic scale visualizes relationship dynamics: red gradients flag positive monotonic links, blue gradients trace inverse associations, and the depth of color saturation corresponds to the mathematical strength of the correlation. Abbreviations: AHI, apnea–hypopnea index; BMI, body mass index; CIMT, carotid intima-media thickness; DBP, diastolic blood pressure; ESS, Epworth Sleepiness Scale; ODI3, 3% oxygen desaturation index; NC, neck circumference; SBP, systolic blood pressure; SOS, Snore Outcomes Survey; SpO_2_, pulse oxygen saturation.

To untangle the exploratory relationships linking continuous CIMT alterations with standard clinical metrics and our targeted sensor data (SSE%-400–496 Hz and SVE%-112–144 Hz), we plotted a comprehensive bivariate correlation matrix (Figure 5A).

Baseline structural assessments revealed that the right CIMT scaled significantly alongside established anthropometric markers of obesity, showing positive linear relationships with both BMI (*r* = 0.29, *p* = 0.043) and NC (*r* = 0.40, *p* = 0.004). Conversely, conventional polysomnographic indices used to score global OSAS severity failed to demonstrate any significant exploratory association with this vascular thickening (all *p* > 0.05).

When evaluating the device-captured biomechanics, both the open-system acoustic and closed-system vibratory metrics yielded distinct phenotypic links to the vascular alterations. The mid-frequency airborne sound signature (SSE%-400–496 Hz) tracked positively with increasing CIMT (*r* = 0.34, *p* = 0.015). Interestingly, this airborne metric remained mathematically isolated; it did not correlate with any demographic traits, physical measurements, or standard sleep lab data.

In contrast, the localized mechanical energy captured by the NPS (SVE%-112–144 Hz) exhibited a highly robust correlation with CIMT expansion (*r* = 0.50, *p* < 0.001). Furthermore, unlike the airborne acoustics, this targeted tissue-vibration metric scaled significantly alongside physical adiposity, demonstrating positive correlations with both BMI (*r* = 0.37, *p* = 0.009) and NC (*r* = 0.28, *p* = 0.048).

Most notably, evaluating the two parallel sensor modalities against one another revealed a lack of statistically significant intersection between the acoustic SSE% and the vibratory SVE% (*r* = 0.24, *p* = 0.096). This mathematical divergence strongly suggests that the airborne sound measured by microphones and the tissue-propagated mechanical stress captured by our piezoelectric sensor captured distinct, potentially complementary aspects of snoring mechanics.

### 3.5. Exploratory Phenotypic Correlations with Categorical Carotid Atherosclerosis

Shifting our focus from continuous intimal expansion to discrete disease states, we generated a secondary exploratory correlation matrix to map clinical, physical, and sensor-derived variables against the categorical presence of right carotid atherosclerosis (Figure 5B).

This targeted analysis revealed that the focal atherosclerotic phenotype scaled positively and significantly with the mid-frequency airborne acoustic signature (SSE%-400–496 Hz; *r* = 0.41, *p* < 0.001). Conversely, within this specific testing cohort, the confirmed presence of carotid plaque exhibited a mathematically inverse relationship with broad overweight or obesity classifications (*r* = −0.41, *p* = 0.003).

Most strikingly, the categorical atherosclerosis profile operated entirely independently of conventional diagnostic expectations. It failed to demonstrate any statistically significant exploratory links with standard polysomnographic severity metrics or classical cardiovascular risk drivers, including advanced age, clinical hypertension, tobacco usage, and hyperlipidemia (all *p* > 0.05).

### 3.6. Exploratory Multivariable Modeling for Right CIMT Phenotypes

To isolate the independent phenotypic value of our bioelectronic device-derived metrics on right CIMT, we engineered a series of hierarchical multivariable linear regression models (Table 3). The computational pipeline sequentially integrated baseline clinical demographics, traditional polysomnographic indices, and the isolated acoustic and vibratory spectral bands (SSE%-404–500 Hz and SVE%-112–144 Hz) flagged during bivariate screening. During model construction, well-established clinical confounders (such as BMI, AHI, and minimum SpO_2_) were properly retained and assessed in sensitivity analyses. Furthermore, ESS score, DBP, AI, and ODI3 were removed to neutralize multicollinearity risks (VIF ≥ 5), and all reported variables maintained a VIF < 5.

The initial baseline configuration (Model 1) captured 31.6% of the phenotypic variance in CIMT (*R*^2^ = 0.316; Cohen’s *f*^2^ = 0.462; *p* = 0.034). In this foundational step, elevated NC surfaced as a significant independent correlate for structural thickening (adjusted *β* = 0.040; 95% CI: 0.005 to 0.074; *p* = 0.027). Integrating standard sleep lab metrics (AHI, mean SpO_2_, and minimum SpO_2_) in Model 2 failed to meaningfully enhance the exploratory explanatory strength (Δ*R*^2^ = 0.019; *p* = 0.786), demonstrating that systemic hypoxia markers do not adequately trace this specific CIMT expansion. NC retained its status as the primary significant marker (adjusted *β* = 0.021; 95% CI: 0.001–0.042; *p* = 0.024).

The fully adjusted framework (Model 3) introduced the targeted bioelectronic device data. Injecting these distinct acoustic and vibratory characteristics dramatically elevated the model’s exploratory power, pushing the total explained variance to 53.3% (*R*^2^ = 0.533) and registering a robust, statistically significant structural upgrade (Δ*R*^2^ = 0.199; Cohen’s *f*^2^ = 0.426; *p* = 0.002). Crucially, the airborne acoustic variable (SSE%-404–500 Hz) emerged as a significant, independent exploratory marker of right CIMT expansion (adjusted *β* = 0.033; 95% CI: 0.007 to 0.059; *p* = 0.021). The tissue-conducted mechanical energy isolated by the NPS (SVE%-112–144 Hz) also displayed a significant positive trajectory (adjusted *β* = 0.021; 95% CI: 0.001 to 0.042; *p* = 0.049). Across all iterations, NC preserved its strong independent association with the vascular phenotype (adjusted *β* = 0.045; 95% CI: 0.012 to 0.079; *p* = 0.010).

To verify the mathematical stability of these findings, we executed a sensitivity analysis omitting two outliers identified by high Cook’s distances. The re-calculated model demonstrated continued robustness (*R*^2^ = 0.626; Δ*R*^2^ = 0.176; Cohen’s *f*^2^ = 0.315; *p* = 0.001). Even after this exclusion, SSE%-404–500 Hz (adjusted *β* = 0.028; 95% CI: 0.005 to 0.052; *p* = 0.019), SVE%-112–144 Hz (adjusted *β* = 0.021; 95% CI: 0.002 to 0.040; *p* = 0.030), and NC (adjusted *β* = 0.050; 95% CI: 0.002 to 0.019; *p* = 0.049) all retained their significance as independent exploratory markers for right CIMT expansion.

### 3.7. Exploratory Multivariable Logistic Modeling for Carotid Atherosclerosis Phenotypes

To map the independent variables associated with the categorical presence of right carotid atherosclerosis, we engineered hierarchical multivariable logistic regression algorithms (Table 4). To address multicollinearity (VIF ≥ 5), NC, ESS, AI, and ODI3 were removed from the model.

The foundational clinical architecture (Model 1) integrated advanced age, biological sex, obesity status, clinical hypertension, tobacco use, hyperlipidemia, and subjective SOS metrics. This baseline configuration failed to isolate any significant independent markers, rendering the overall model statistically uninformative (Omnibus *p* = 0.789; Nagelkerke *R*^2^ = 0.103). Introducing the AHI, mean SpO_2_, and minimum SpO_2_ in Model 2 provided no measurable diagnostic upgrade; all covariates remained non-significant, and the systemic hypoxic marker failed to improve the overarching model fit (Omnibus *p* = 0.673).

However, integrating the bioelectronic device-derived spectral metrics into the fully adjusted framework (Model 3) radically transformed its explanatory capacity (Omnibus *p* = 0.044; Nagelkerke *R*^2^ = 0.433). Within this optimized algorithm, the mid-frequency airborne acoustic signature (SSE%-404–500 Hz) surfaced as a highly significant, independent exploratory marker for the atherosclerotic phenotype. Quantitatively, every 1% expansion in this specific acoustic energy band is associated with an elevated odds of presenting with focal carotid plaque (aOR = 1.828; 95% CI: 1.160 to 2.882; *p* = 0.009).

In contrast, the localized mechanical vibration (SVE%-112–144 Hz) captured by the piezoelectric sensor did not demonstrate a statistically significant exploratory link to this categorical plaque formation outcome (aOR = 1.192; 95% CI: 0.796 to 1.785; *p* = 0.394). Throughout this final iteration, both classical cardiovascular risk factors and profound hypoxic dips remained entirely non-significant.

The structural integrity of this final multivariable framework was corroborated by the Hosmer-Lemeshow goodness-of-fit test (*p* = 0.396). Additionally, variance inflation factors for all integrated parameters remained safely suppressed (maximum VIF = 3.07 for minimum SpO_2_), validating the absence of multicollinearity and verifying the mathematical stability of our exploratory regression outputs.

To assess the stability of the regression estimates, a sensitivity analysis was performed after removing two observations with markedly elevated Cook’s distances. The revised model remained statistically robust (Omnibus *p* = 0.049; Nagelkerke *R*^2^ = 0.462). Notably, even after excluding these influential cases, SSE%-404–500 Hz continued to exhibit a significant independent association with right-sided CIMT enlargement (aOR = 1.818; 95% CI: 1.109–2.983; *p* = 0.018).

### 3.8. Diagnostic Performance and Exploratory Modeling for Carotid Atherosclerosis

**Figure 6:**
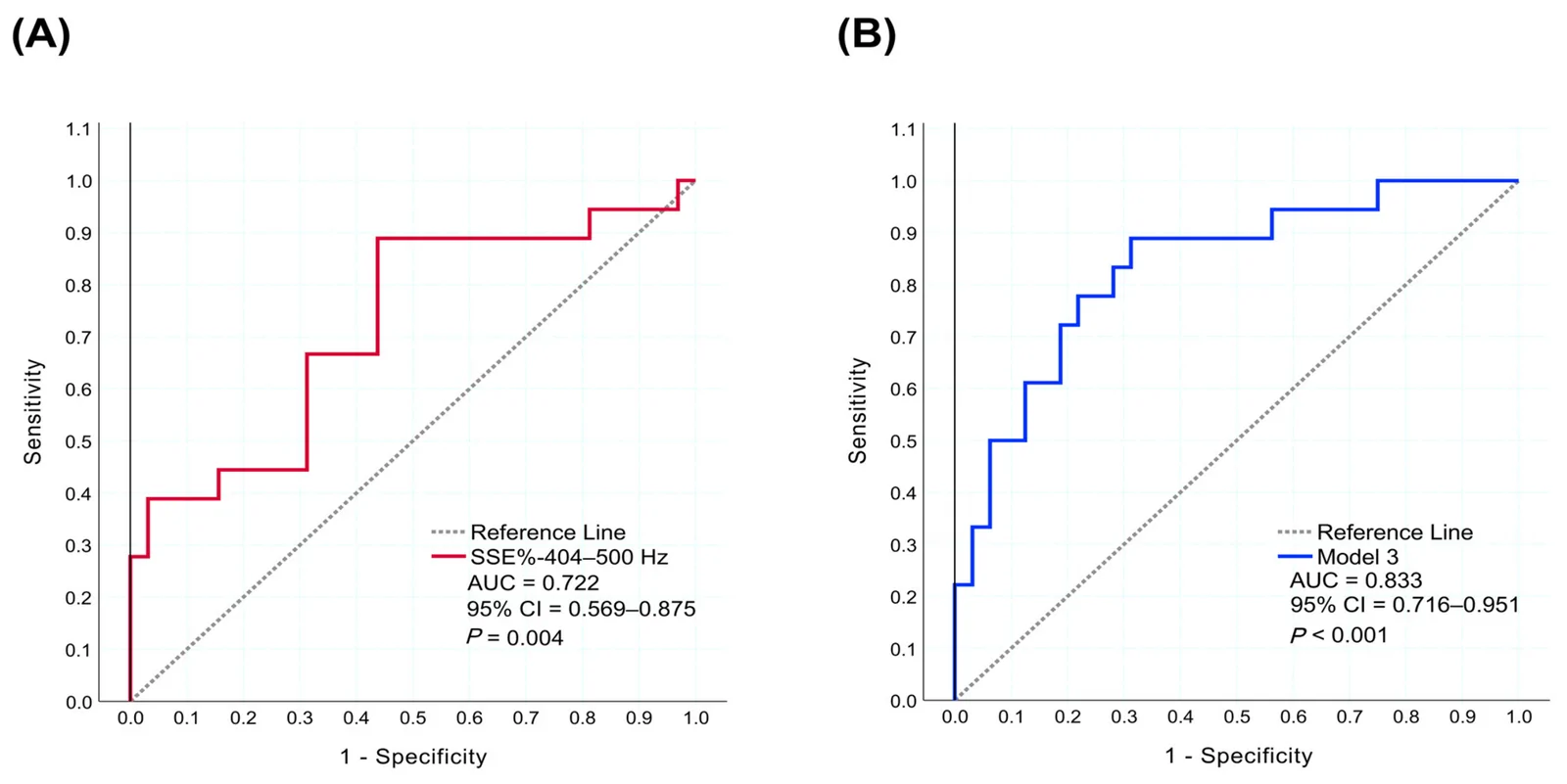
Receiver operating characteristic (ROC) curves mapping the exploratory diagnostic utility of the acoustic sensor data for focal carotid atherosclerosis. (**A**) Exploratory capacity of the standalone acoustic signature. The isolated mid-frequency airborne energy (SSE%-404–500 Hz) demonstrates a significant baseline capability to classify the atherosclerotic phenotype, yielding an area under the curve (AUC) of 0.722 (95% CI: 0.569–0.875; *p* = 0.004). (**B**) Enhanced algorithmic classification via the integrated multivariable framework (Model 3). Computationally fusing the patient’s baseline clinical profile with the targeted acoustic sensor output substantially upgrades the model’s exploratory diagnostic power, driving the AUC to 0.833 (95% CI: 0.716–0.951; *p* < 0.001). Abbreviations: AUC, area under the ROC curve; CI, confidence interval; SSE%, normalized frequency-domain snoring sound energy.

To quantify the diagnostic utility of our targeted sensor outputs, we executed ROC curve algorithms. Evaluating the mid-frequency airborne acoustic signature (SSE%-404–500 Hz) in isolation revealed a statistically significant exploratory capacity to detect the focal carotid atherosclerosis phenotype. This single bioelectronic marker generated an area under the curve (AUC) of 0.722 (95% CI: 0.569–0.875; *p* = 0.004) (Figure 6A). By calibrating the diagnostic threshold to an optimal energy cutoff of 1.95%, this standalone acoustic parameter delivered an overall screening accuracy of 68%, driven by a robust sensitivity of 89% alongside a 56% specificity.

We subsequently tested the exploratory predictive bounds of the fully adjusted multivariable logistic architecture (Model 3), which computationally fuses the targeted acoustic sensor data with the patients’ baseline clinical profiles (Figure 6B). This integrated bioelectronic and clinical model substantially upgraded the diagnostic performance, expanding the AUC to 0.833 (95% CI: 0.716–0.951; *p* < 0.001). Operating at an optimized exploratory probability threshold of 0.26, the comprehensive multivariable framework achieved a global accuracy of 76%. Crucially, it preserved the high 89% sensitivity while significantly elevating diagnostic specificity to 69%.

## 4. Discussion

### 4.1. Clinical Imperatives and Bioelectronic Solutions in Snoring Assessment

OSAS remains a pervasive catalyst for severe cardiovascular complications, yet it is chronically underdiagnosed because traditional in-laboratory polysomnography is an unscalable, resource-heavy bottleneck [42,43]. This diagnostic void underscores an urgent clinical mandate for deployable wearable physical technologies and home-based bioelectronic screening [44].

However, extracting reliable cardiovascular risk metrics from snoring introduces profound physical challenges. Conventional ambient microphones operate as open systems, making them highly vulnerable to spatial displacement and environmental acoustic pollution [11,44]. More critically, airborne recordings only capture the auditory byproduct of the respiratory struggle, severely underestimating the actual biomechanical force striking the cervical tissues [45]. Prior experimental data highlights this physical discrepancy, revealing that the true mechanical energy absorbed by the carotid artery can exceed the airborne acoustic energy captured by room microphones by a magnitude of 17,000 [46].

To circumvent the inherent flaws of open-air audio recording and the invasiveness of experimental animal models [9,46], modern bioelectronics must pivot to closed-system architectures. By securing directly to the epidermis, the neck-wearable piezoelectric sensor acts as a proxy for tissue-conducted mechanical energy assaulting the carotid wall, rather than measuring the ‘true’ energy reaching the artery itself, since it measures from the skin surface [17]. This surface-coupled approach intrinsically safeguards patient privacy by discarding audible conversation in favor of mechanical strain data [5]. Ultimately, mapping how these specific, localized mechanical frequencies correlate with human endothelial changes is the foundational step for elevating portable sensors into precision exploratory tools for cardiovascular diagnostics [9].

### 4.2. Principal Findings and Bioelectronic Implications

This investigation mapped the exploratory relationships linking distinct spectral characteristics of snoring—captured via both airborne acoustics and tissue-coupled vibrations—to subclinical architectural changes in the right carotid artery. The central observation is that highly localized frequency bands act as independent exploratory phenotypes for vascular alteration, decoupling entirely from standard systemic hypoxia or clinical severity indices.

#### 4.2.1. Mechanical Vibration and Intimal Expansion Phenotypes

Elevated tissue-conducted energy within the 112–144 Hz bandwidth (SVE%-112–144 Hz) demonstrated a robust exploratory correlation with subclinical intimal thickening. Operating alongside NC—an established surrogate for localized peripharyngeal adiposity [8,47]—this discrete mechanical signal surfaced as a significant independent phenotypic marker for right CIMT.

Historically, cardiovascular decline in OSAS is framed around global hypoxemia and autonomic surges [48,49,50]. However, our sensor data aligns with a localized biomechanical paradigm [9,50]. As shown in our spectral distribution, the vast majority of absolute raw energy exists in the ultra-low frequencies (e.g., 4–36 Hz), which primarily reflects gross aerodynamic tissue displacement. Experimental models suggest that as mechanical oscillations reach the carotid lumen, the nonhomogeneous arterial wall naturally amplifies frequencies between 75 and 275 Hz due to its natural resonance frequency window [46]. Therefore, even though our isolated 112–144 Hz band accounts for a tiny fraction of the total energy, it falls squarely within this resonance window, representing a state of peak physical strain that exerts disproportionately high mechanical stress on the local endothelium. We hypothesize that chronic exposure to this localized vibratory stress correlates with structural remodeling phenotypes by potentially disrupting endothelial permeability [9,15] and vasomotor homeostasis [9]. Consequently, capturing this specific mechanical bandwidth provides a powerful exploratory metric for monitoring subclinical vascular progression without requiring definitive causal assumptions.

#### 4.2.2. Airborne Acoustics, Intimal Expansion Phenotypes, and the Atherosclerotic Profile

In addition to the tissue-conducted mechanical data, the mid-frequency airborne acoustic signature (SSE%-404–500 Hz) emerged as a significant, independent exploratory marker for both continuous right CIMT expansion and the categorical presence of carotid plaque. The anatomical generation of this sound provides context for these specific exploratory links:

1. Spatial Proximity: Mid-frequency acoustic peaks (centering near 490 Hz) primarily originate from obstructions in the lower airway, specifically involving the epiglottis [7,51,52]. Crucially, these lower pharyngeal structures sit immediately adjacent to the carotid bifurcation, the anatomical zone most susceptible to early intimal thickening and focal plaque accumulation [53].
2. Biomechanical Transmission: Due to this intimate anatomical proximity, concentrated acoustic energy spanning 404–500 Hz propagates directly toward the adjacent vascular wall. We theorize that this transmission acts as a localized physical stressor. The resulting rapid vibratory oscillations may disturb normal hemodynamics [15], potentially serving as an environmental correlate for the localized inflammation and oxidative stress observed in early atherogenesis.
3. Surface Acoustic Wave (SAW) Dynamics: It is further hypothesized that the intense 404–500 Hz energy functions dynamically as a surface acoustic wave [14]. In fluid mechanics, SAWs induce localized micro-fluidic actuations that alter binding kinetics. Within the biological context, this phenomenon might theoretically facilitate the receptor-mediated endocytosis of low-density lipoproteins across a compromised endothelium [54]. This SAW-mediated LDL internalization is a proposed hypothesis drawn from in vitro and animal experimental models; direct in vivo mechanistic proof in humans remains an area for future research. Therefore, an elevated SSE%-404–500 Hz operates as a highly specific exploratory marker, flagging the high-intensity, localized physical stressors associated with both continuous intimal expansion and focal plaque deposition.

#### 4.2.3. Algorithmic Synergy and Exploratory Diagnostic Predictive Capability

In isolation, the targeted mid-frequency airborne signature (SSE%-404–500 Hz) exhibited a statistically significant exploratory capacity to classify the carotid atherosclerosis phenotype. However, this exploratory marker scaled considerably when the discrete bioelectronic data was algorithmically fused with the patients’ baseline clinical profiles. This multimodal synergy mirrors the highly complex, multi-variable architecture of cardiovascular disease:

1. Systemic Preconditioning: Traditional clinical baselines (such as biological sex, physical adiposity, hypertension, and dyslipidemia) effectively map the generalized, pro-inflammatory vulnerability of the vascular network. Yet, these systemic biochemical metrics remain anatomically agnostic; they lack the spatial resolution required to predict the focal distribution of plaque accumulation [55].
2. The Localized Biomechanical Correlate: Conversely, the targeted acoustic metric (SSE%-404–500 Hz) functions as a highly specific phenotypic proxy, representing the localized physical stressors concentrated immediately adjacent to the vulnerable carotid bifurcation [7,14].

By computationally uniting a patient’s broad biochemical susceptibility with this discrete, highly localized biomechanical indicator, the integrated framework substantially upgrades overall exploratory marker precision. This algorithmic fusion establishes high-resolution bioelectronic sensor data as a formidable, non-invasive screening tool with the potential capability to assist in stratifying stroke risk by mapping the specific exploratory phenotypes associated with localized structural alterations.

#### 4.2.4. Divergence from Conventional Polysomnography

Crucially, the standard polysomnographic indices traditionally utilized to quantify global OSAS severity—specifically AHI, AI, ODI3, and systemic SpO_2_ profiles—failed to demonstrate any significant exploratory associations with either continuous CIMT expansion or the categorical atherosclerotic phenotype. Furthermore, cross-evaluating the targeted airborne acoustic data against the tissue-coupled vibratory metrics revealed a non-significant correlation. This divergence indicates that the ambient microphone and the piezoelectric sensor capture distinct, potentially complementary aspects of snoring mechanics. Rather than relying on traditional, systemic hypoxia-centric diagnostic models, these findings demonstrate that discrete acoustic and mechanical energies provide independent bioelectronic signatures, each mapping to specific, localized structural vascular phenotypes.

### 4.3. Methodological Evolution of Snoring Assessment Paradigms

Table 5 traces the technological paradigm shifts in characterizing the exploratory associations between upper airway snoring dynamics and subclinical carotid vascular phenotypes. This clinical evolution reflects a steady transition from subjective reporting toward localized, high-fidelity bioelectronic tracking.

#### 4.3.1. Subjective and Categorical Screening

Early epidemiological frameworks heavily leveraged self-administered patient questionnaires to screen for snoring behaviors [10,11,55,56,57,58]. While instrumental in establishing initial clinical associations, these subjective instruments are inherently restricted by retrospective recall bias. Furthermore, they compress complex, continuous physiological behaviors into coarse, nominal data points, completely failing to capture or quantify the actual physical forces interacting with peripheral neck structures. This lack of objective quantification explains why several previous questionnaire-based studies reported no significant association between snoring and CIMT; self-reporting simply cannot capture the precise physical intensity and dose–response required to track localized vascular remodeling.

#### 4.3.2. Macro-Polysomnographic Metrics

Subsequent clinical research advanced to objective, lab-based telemetry, integrating time- and event-dependent sleep parameters such as the snoring index or cumulative snoring duration [50,59]. Although these metrics standardized measurement consistency, they remain fundamentally blind to the underlying physics of upper airway collapse. They track macroscopic event frequencies and durations but cannot evaluate the specific amplitude, spectral composition, or mechanical energy profiles interfacing with the adjacent carotid wall.

#### 4.3.3. Open-System Acoustic Analytics

Recent innovations in digital signal processing introduced high-resolution ambient sound analysis, confirming that discrete airborne acoustic signatures correlate independently with continuous CIMT expansion and focal stenosis [7,14,60]. However, previous studies have identified vastly different frequency bands associated with vascular risk. This spectral heterogeneity is largely driven by variations in equipment—such as the use of ambient microphones placed at varying distances versus direct-contact sensors—as well as inherent differences in the anatomical source of snoring among distinct patient cohorts. Despite this exploratory marker, relying solely on room-mounted microphones introduces severe physical and environmental limitations. Operating as open systems, ambient microphones are highly susceptible to background acoustic interference, room reverberation, and changes in patient sleep posture. More critically, airborne audio data only intercepts the acoustic energy escaping into the room, which vastly underestimates the true internal, tissue-propagated physical forces.

#### 4.3.4. Dual-Modality Closed-System Architecture

The bioelectronic framework presented in this study addresses these diagnostic blind spots by deploying a synchronized, dual-sensor modality. By simultaneously tracking localized, tissue-conducted mechanical energy via a surface-coupled piezoelectric sensor (112–144 Hz) and high-fidelity airborne sound through an acoustic channel (404–500 Hz), this architecture successfully isolates distinct mechanotransduction phenotypes. Operating as a closed system over the carotid triangle, this integrated configuration provides a precise, non-invasive, and artifact-resistant exploratory framework for screening regional vascular phenotypes, establishing a clear technological advancement over traditional clinical metrics and isolated ambient recording.

**Table 5:** Summary of literature evaluating the association between snoring measurements and the risk of increased carotid intima-media thickness (CIMT) or carotid atherosclerosis.

Study (Year)	Study Population	Snoring Measurement Method	Key Findings Regarding CIMT and Carotid Atherosclerosis
Lee et al. (2008) [50]	110 volunteers (mild, nonhypoxic OSAS)	Objective: Polysomnography (snoring sleep time %)	Substantial nocturnal snoring duration (>50% of sleep) independently correlated with carotid atherosclerosis (OR = 10.5), while showing no association with femoral vascular phenotypes.
Ramos-Sepulveda et al. (2010) [56]	1605 community cohort (Northern Manhattan)	Subjective: Self-reported frequency (>4 times/week)	Habitual snoring failed to demonstrate an independent association with CIMT expansion upon adjusting for baseline cardiovascular variables.
Li et al. (2012) [55]	1050 urban Chinese adults (aged 50–79)	Subjective: Self-reported frequency (≥5 days/week)	Self-reported habitual snoring exhibited a significant exploratory link to both structural CIMT thickening (OR = 1.71) and focal carotid bifurcation plaque (OR = 3.63).
Apaydin et al. (2013) [47]	87 patients referred for sleep evaluation	Objective: Polysomnography (habitual simple snoring vs. OSAS)	Patients exhibiting OSAS demonstrated significantly greater structural CIMT expansion (0.75 mm) compared to the simple habitual snoring cohort (0.65 mm).
Kim et al. (2014) [10]	3129 prospective cohort (Korea, 4-year follow-up)	Subjective: Self-reported frequency	Baseline habitual snoring in female subjects correlated with elevated CIMT risk, though longitudinal tracking revealed no accelerated progression of subclinical plaque over a 4-year interval.
Lee et al. (2014) [57]	7330 community cohort (Korea)	Subjective: Self-reported frequency	Subjective snoring correlated with elevated CIMT measurements (0.726 vs. 0.713 mm) and higher odds for structural thickening (OR = 1.25), yet lacked association with focal plaque formation.
Salepci et al. (2015) [59]	102 patients evaluated for SDB	Objective: Polysomnography (snoring index)	Objective snoring indices scaled significantly alongside structural CIMT expansion, demonstrating robust phenotypic correlations with both snoring intensity and global OSAS severity.
Lee et al. (2016) [14]	30 newly diagnosed OSAS patients	Objective: Acoustic sound analysis (frequency and energy)	High-resolution acoustic mapping identified significant exploratory correlations between CIMT and specific spectral sound energies (0–20 Hz and 652–1500 Hz).
Kirkham et al. (2017) [11]	133 subjects with asymptomatic carotid disease	Subjective: Self-reported frequency and loudness	Subjective snoring metrics linked independently to high-risk structural plaque phenotypes on MRI, including fibrous cap rupture (OR = 4.4) and intraplaque hemorrhage (OR = 8.2).
Kim et al. (2017) [60]	180 non-apneic participants	Objective: Microphone (snoring time)	Objective acoustic tracking in female cohorts revealed that progressive CIMT thickening scales positively with cumulative nocturnal snoring duration.
Ghofraniha et al. (2017) [58]	80 patients with Type 2 diabetes	Subjective: Self-reported snoring	Diabetic cohorts reporting subjective snoring exhibited significantly more pronounced CIMT structural expansion (0.72 mm) relative to non-snorers (0.56 mm).
Chuang et al. (2021) [7]	70 early OSA patients	Objective: Acoustic (normalized snoring sound energy)	Discrete airborne acoustic energies served as distinct phenotypic markers; 301–850 Hz correlated with continuous CIMT alterations, whereas 4–300 Hz linked to focal carotid stenosis.
Görgülü et al. (2025) [8]	140 patients with atherosclerosis	Objective: Polysomnography (primary snoring)	Polysomnographic primary snoring exhibited a robust, localized association with expanded CIMT (0.90 vs. 0.65 mm) without impacting femoral IMT, supporting a localized biomechanical lin.

Abbreviations: CIMT, carotid intima-media thickness; MRI, magnetic resonance imaging; OR, odds ratio; OSAS, obstructive sleep apnea syndrome; SDB, sleep-disordered breathing.

### 4.4. Constraints and Future Directions for Bioelectronic Validation

While this analysis identifies exploratory phenotypes linking discrete spectral snoring signatures to subclinical vascular alterations, these preliminary findings must be viewed through a set of methodological constraints.

First, the cross-sectional architecture precludes any definitive assessment of longitudinal causation. The identified spectral correlations—specifically SVE%-112–144 Hz and SSE%-404–500 Hz—serve as phenotypic markers of vascular structural states rather than proven drivers of plaque progression.

Second, a critical statistical limitation of this pilot study is the approach to frequency band selection. The specific targeted bands (SSE%-404–500 Hz and SVE%-112–144 Hz) were not pre-specified but were identified post hoc through a full-spectrum high-resolution scan. Because we did not apply multiple testing corrections (such as false discovery rate or permutation testing), and because we lacked cross-validation due to our small sample size, there is a substantially inflated risk of Type I errors (false-positive findings) [61]. Consequently, our findings must be interpreted strictly as exploratory associations and hypothesis-generating. The specifically identified bands must be viewed with caution until they are externally validated in larger, independent cohorts using rigorous cross-validation.

Third, the broad generalizability of our results is constrained by the small cohort size (*n* = 50) and a specific demographic skew toward middle-aged men (84%) with severe OSAS. Because anatomical differences between sexes—particularly regarding neck fat distribution and the spatial relationship between the upper airway and carotid arteries—could substantially modify the transmission of mechanical vibrations [62], these phenotypic models require further validation in female cohorts and in patients spanning the full spectrum of SDB severity. Moreover, our restricted sub-cohort dimensions inevitably invite extreme sampling error, which likely generated clinical anomalies in our baseline data. Most notably, the counterintuitive drop in hypertension prevalence observed in the severe atherosclerosis group relative to the intermediate thick CIMT group is almost certainly a statistical artifact driven by this limitation. Consequently, extended research in primary snorers without OSA and in broader community populations is essential to confirm whether these localized mechanotransduction effects persist without the confounding presence of severe intermittent hypoxia.

Fourth, our vascular assessments were localized to the right carotid artery. While this remains the standard clinical site for evaluating systemic subclinical atherosclerosis [63], a unilateral scanning protocol may miss bilateral architectural disparities or the full extent of cervical disease. Furthermore, the absence of continuous nocturnal hemodynamic tracking (e.g., beat-to-beat pressure monitoring) leaves a gap in understanding the temporal interplay between localized mechanical stress and transient nocturnal hypertensive surges.

Furthermore, a significant methodological constraint of this pilot study is that the exploratory multivariable models were developed and evaluated within the same small cohort, which included only 18 positive atherosclerosis cases. This introduces a severe risk of model overfitting, meaning the reported diagnostic capacity and AUC values are likely overestimations [64]. While methods such as penalized regression or bootstrap validation would ideally mitigate this risk, our extremely limited sample size makes such internal validation highly unstable. Consequently, rigorous external validation in larger, independent cohorts is strictly required before these proposed bioelectronic markers can be considered for clinical translation.

To bridge these gaps, future research should transition to large-scale, multi-center longitudinal designs. These studies are essential for tracking the natural trajectory of intimal thickening and plaque vulnerability, testing whether chronic exposure to high-risk spectral frequencies correlates with subsequent structural remodeling. Future investigations must also prioritize diverse participant representation and incorporate broader clinical phenotypes. Finally, interventional trials are needed to determine if targeted therapeutic interventions—such as positive airway pressure therapy, custom oral appliances, or airway surgery—can measurably attenuate these specific energy bands and, by extension, modify the progression of carotid atherosclerosis. Validating this feedback loop is the necessary next step for translating these exploratory bioelectronic metrics into established clinical practice.

To further enhance sensor reliability for long-term wearable monitoring, future hardware iterations of our neck sensor could leverage recent advancements in flexible bioelectronic materials. For instance, incorporating microstructured pressure sensing and rigid-flexible hybrid designs can significantly broaden the operating range and accelerate dynamic response times [65]. Additionally, the implementation of cross-scale wrinkled structures and bioinspired wrinkle-crack interactions has demonstrated exceptional capabilities in maintaining ultrahigh linearity and ultralow hysteresis, effectively mitigating measurement errors caused by unavoidable pre-strain during conformal sensor attachment on human skin [66,67]. Finally, integrating robust environmental protection strategies—such as elastic silicone tube encapsulation—can safeguard signal fidelity against external interferences like moisture and dust, ensuring sustained stability and patient comfort during continuous real-world tracking [68].

## 5. Conclusions

This research introduces a refined bioelectronic paradigm that shifts the focus of sleep medicine from counting respiratory events to mapping the physical forces acting upon the human vasculature. By deploying synchronized piezoelectric and acoustic sensing, we have demonstrated that specific frequency-domain signatures act as granular phenotypic markers for structural carotid damage.

Our findings indicate that localized SVE%-112–144 Hz and airborne SSE%-404–500 Hz offer high-fidelity insights into vascular health that remain invisible to standard polysomnographic screening. Because these metrics operate independently of systemic hypoxia markers like the AHI, they reveal a previously underappreciated pathway of potential mechanical associations. By characterizing snoring as a spectrum of localized biomechanical energy rather than a binary symptom, this dual-modality approach provides a transformative instrument for precision risk stratification. Ultimately, this framework moves the field toward a more nuanced, individualized understanding of how nocturnal airway dynamics dictate long-term cardiovascular outcomes.

## Figures and Tables

**Table 1:** Characteristics of the study population and comparisons among participants with different carotid artery profiles.

Characteristics	All Participants	Normal CIMT	Thick CIMT	Carotid Atherosclerosis	*p*-Value ^a^
	(*n* = 50)	(*n* = 17)	(*n* = 15)	(*n* = 18)	
Clinical parameters					
Age, y	39.7 ± 9.5	39.5 ± 10.5	38.7 ± 7.8	40.8 ± 10.1	0.816
Male, *n* (%)	42 (84)	16 (94)	13 (87)	13 (72)	0.199
BMI, kg/m^2^	28.39 ± 3.91	26.98 ± 4.13	29.50 ± 3.95	28.80 ± 3.44	0.166
NC, cm	39.7 ± 2.5	39.0 ± 1.8	40.9 ± 3.0	39.4 ± 2.4	0.070
SBP, mmHg	127.2 ± 16.5	119.6 ± 10.7 ^b^	137.5 ± 16.4 ^b^	125.8 ± 17.5	**0.006**
DBP, mmHg	75.4 ± 11.3	71.6 ± 8.6	80.8 ± 11.2	78.1 ± 12.8	0.100
ESS, score	14 (10–18)	13 (10–17)	14 (9–20)	14 (10–18)	0.963
SOS, score	36.1 ± 10.7	39.2 ± 10.7	33.6 ± 10.2	35.2 ± 11.1	0.316
Traditional atherosclerotic cardiovascular disease risk factors					
Increased age, *n* (%)	13 (26)	4 (24)	5 (33)	4 (22)	0.738
Overweight or obesity, *n* (%)	45 (90)	14 (82)	14 (93)	17 (94)	0.431
Cigarette smoking, *n* (%)	13 (26)	6 (35)	3 (20)	4 (22)	0.555
Hypertension, *n* (%)	9 (18)	0 (0)	6 (40)	3 (17)	**0.013**
DM, *n* (%)	0 (0)	0 (0)	0 (0)	0 (0)	–
Hyperlipidemia, *n* (%)	13 (26)	2 (12)	5 (33)	6 (33)	0.258
Polysomnographic parameters					
AHI, events/h	66.0 (26.4–80.2)	66.4 (18.7–75.4)	70.5 (38.0–83.6)	43.6 (19.3–83.9)	0.256
ODI3, events/h	46.1 (19.4–69.4)	53.6 (10.4–69.2)	62.7 (25.2–72.6)	29.4 (11.8–64.4)	0.173
Mean SpO_2_, %	95 (93–95)	94 (93–95)	94 (92–95)	95 (94–95)	0.330
Minimal SpO_2_, %	81.1 ± 8.3	81.6 ± 6.3	77.8 ± 10.1	83.5 ± 7.8	0.139

Continuous data are presented as means ± standard deviations or medians (interquartile ranges), as appropriate; categorical data are expressed as absolute frequencies (percentages). ^a^ Differences among groups were evaluated using a one-way analysis of variance for normally distributed means, the Kruskal–Wallis test for medians, and the χ^2^ test for categorical percentages. ^b^ Significant post hoc pairwise differences were adjusted using the Bonferroni correction. Bold *p*-values indicate statistical significance (*p* < 0.05). Abbreviations: AHI, apnea–hypopnea index; BMI, body mass index; CIMT, carotid intima-media thickness; DBP, diastolic blood pressure; DM, diabetes mellitus; ESS, Epworth Sleepiness Scale; NC, neck circumference; ODI3, 3% oxygen desaturation index; SBP, systolic blood pressure; SOS, Snore Outcomes Survey; SpO_2_, pulse oxygen saturation.

**Table 2:** Snoring characteristics of the study population and comparisons among participants with different carotid artery profiles.

Characteristics	All Participants	Normal CIMT	Thick CIMT	Carotid Atherosclerosis	*p*-Value ^a^
	(*n* = 50)	(*n* = 17)	(*n* = 15)	(*n* = 18)	
Snoring sound analysis					
SSE%-4–100 Hz, %	66.35 ± 21.73	69.29 ± 24.76	67.77 ± 19.91	60.38 ± 20.75	0.624
SSE%-104–200 Hz, %	9.26 ± 8.06	8.48 ± 8.73	9.46 ± 8.56	9.82 ± 7.35	0.885
SSE%-204–300 Hz, %	5.83 ± 4.76	5.31 ± 4.25	4.69 ± 4.03	7.27 ± 5.61	0.263
SSE%-304–400 Hz, %	3.23 ± 3.68	3.34 ± 4.79	3.03 ± 2.90	3.28 ± 3.25	0.970
SSE%-404–500 Hz, %	2.47 ± 1.97	1.62 ± 1.49 ^b^	2.17 ± 1.48	3.53 ± 2.29 ^b^	**0.010**
SSE%-504–600 Hz, %	2.55 ± 2.50	2.23 ± 2.15	2.03 ± 1.51	3.30 ± 3.29	0.280
SSE%-604–700 Hz, %	2.66 ± 2.77	2.72 ± 3.46	2.56 ± 2.84	2.68 ± 2.04	0.987
SSE%-704–800 Hz, %	1.34 ± 1.12	1.25 ± 1.34	1.27 ± 1.02	1.48 ± 1.02	0.807
SSE%-804–900 Hz, %	0.83 ± 0.79	0.68 ± 0.65	0.73 ± 0.79	1.06 ± 0.90	0.305
SSE%-904–1000 Hz, %	0.90 ± 0.94	0.69 ± 0.67	0.98 ± 1.18	1.05 ± 0.93	0.507
SSE%-1004–1100 Hz, %	0.78 ± 1.11	0.52 ± 0.46	1.177 ± 1.67	0.70 ± 0.90	0.231
SSE%-1104–1200 Hz, %	0.85 ± 1.54	0.90 ± 1.19	0.64 ± 0.68	0.97 ± 2.25	0.823
SSE%-1204–1300 Hz, %	1.02 ± 1.77	1.55 ± 2.60	0.48 ± 0.36	0.96 ± 1.44	0.234
SSE%-1304–1400 Hz, %	0.90 ± 1.85	0.97 ± 2.06	0.76 ± 1.51	0.96 ± 1.99	0.940
SSE%-1404–1500 Hz, %	1.03 ± 4.14	0.45 ± 1.10	2.25 ± 7.34	0.56 ± 1.39	0.402
Snoring vibration analysis					
SVE%-4–36 Hz, %	93.72 ± 17.81	90.55 ± 24.35	98.52 ± 2.45	92.72 ± 17.87	0.440
SVE%-40–72 Hz, %	1.71 ± 3.93	2.42 ± 5.37	0.49 ± 0.77	2.06 ± 3.88	0.353
SVE%-76–108 Hz, %	0.93 ± 3.38	0.72 ± 1.29	0.23 ± 0.38	1.71 ± 5.48	0.443
SVE%-112–144 Hz, %	0.71 ± 2.62	0.31 ± 0.79	0.13 ± 0.19	1.58 ± 4.23	0.211
SVE%-148–180 Hz, %	0.43 ± 1.62	0.41 ± 1.06	0.11 ± 0.18	0.72 ± 2.52	0.568
SVE%-184–216 Hz, %	0.37 ± 1.51	0.39 ± 1.20	0.08 ± 0.15	0.59 ± 2.24	0.627
SVE%-220–252 Hz, %	0.25 ± 1.05	0.37 ± 1.41	0.05 ± 0.10	0.29 ± 1.12	0.685
SVE%-256–288 Hz, %	0.20 ± 0.98	0.42 ± 1.62	0.04 ± 0.08	0.13 ± 0.47	0.530
SVE%-292–324 Hz, %	0.18 ± 1.07	0.47 ± 1.83	0.04 ± 0.07	0.02 ± 0.06	0.398
SVE%-328–360 Hz, %	0.20 ± 1.19	0.52 ± 2.05	0.04 ± 0.08	0.02 ± 0.06	0.398
SVE%-364–396 Hz, %	0.22 ± 1.32	0.58 ± 2.26	0.05 ± 0.09	0.02 ± 0.07	0.397
SVE%-400–432 Hz, %	0.24 ± 1.44	0.63 ± 2.47	0.05 ± 0.10	0.03 ± 0.08	0.396
SVE%-436–468 Hz, %	0.26 ± 1.57	0.68 ± 2.69	0.05 ± 0.11	0.03 ± 0.08	0.396
SVE%-472–504 Hz, %	0.28 ± 1.69	0.74 ± 2.90	0.06 ± 0.12	0.03 ± 0.09	0.396

Continuous data are presented as means ± standard deviations. ^a^ Differences among groups were evaluated using a one-way analysis of variance for normally distributed means. ^b^ Significant post hoc pairwise differences were adjusted using the Bonferroni correction. Bold *p*-values indicate statistical significance (*p* < 0.05). Abbreviations: CIMT, carotid intima-media thickness; SSE%, normalized snoring sound energy; SVE%, normalized snoring vibration energy.

**Table 3:** Hierarchical multivariable linear regression models identifying independent variables associated with right carotid intima-media thickness (CIMT).

Variables	Model 1			Model 2			Model 3		
	*β* (95% CI)	*p*-Value	VIF	*β* (95% CI)	*p*-Value	VIF	*β* (95% CI)	*p*-Value	VIF
Clinical parameters									
Age, y	0.001 (−1.701 to 0.279)	0.155	1.19	0.001 (−0.005 to 0.008)	0.702	1.32	0.001 (−0.005 to 0.006)	0.862	1.40
Male sex	−0.175 (−0.360 to 0.011)	0.064	1.80	−0.176 (−0.367 to 0.016)	0.071	1.82	−0.165 (−0.335 to 0.004)	0.056	1.93
BMI, kg/m^2^	−0.007 (−0.028 to 0.014)	0.524	2.63	−0.008 (−0.030 to 0.014)	0.463	2.69	−0.016 (−0.036 to 0.003)	0.099	2.84
NC, cm	0.040 (0.005 to 0.074)	** 0.027 **	2.95	0.043 (0.006 to 0.081)	** 0.024 **	3.20	0.045 (0.012 to 0.079)	** 0.010 **	3.52
SBP, mmHg	0.001 (−0.002 to 0.005)	0.406	1.24	0.002 (−0.002 to 0.006)	0.327	1.49	0.002 (−0.002 to 0.005)	0.275	1.50
Tobacco use	−0.014 (−0.140 to 0.111)	0.820	1.18	−0.026 (−0.171 to 0.118)	0.714	1.49	−0.058 (−0.184 to 0.068)	0.356	1.51
Hyperlipidemia	0.063 (−0.060 to 0.187)	0.307	1.14	0.059 (−0.077 to 0.196)	0.385	1.33	0.024 (−0.096 to 0.144)	0.689	1.39
SOS, score	−0.003 (−0.060 to 0.187)	0.254	1.04	−0.003 (−0.008 to 0. 002)	0.233	1.13	−0.007 (−0.008 to 0.002)	0.214	1.14
Polysomnographic parameters									
AHI, events/h	–	–	–	−0.001 (−0.003 to 0.002)	0.684	2.75	−0.001 (−0.003 to 0.002)	0.715	2.87
Mean SpO_2_, %	–	–	–	−0.003 (−0.039 to 0.032)	0.853	3.02	−0.010 (−0.042 to 0.022)	0.532	3.27
Minimum SpO_2_, %	–	–	–	0.003 (−0.008 to 0.013)	0.644	2.97	0.003 (−0.006 to 0.013)	0.468	2.98
Snoring characteristics									
SSE%-404–500 Hz, %	–	–	–	–	–	–	0.033 (0.007 to 0.059)	** 0.014 **	1.24
SVE%-112–144 Hz, %	–	–	–	–	–	–	0.021 (0.001 to 0.042)	** 0.049 **	1.47
Model summary									
*R* ^2^	0.316			0.334			0.533		
Δ*R*^2^	0.316			0.019			0.199		
Cohen’s *f*^2^	0.462			0.029			0.426		
*p*-value for Δ*R*^2^	** 0.034 **			0.786			** 0.002 **		

Epworth Sleepiness Scale score, diastolic blood pressure, apnea index, and 3% oxygen desaturation index were removed to neutralize multicollinearity risks (VIF ≥ 5). Bold *p*-values indicate statistical significance (*p* < 0.05). Abbreviations: CI, confidence interval; NC, neck circumference; SBP, systolic blood pressure; SOS, Snore Outcomes Survey; SSE%, normalized frequency-domain snoring sound energy; SVE%, normalized snoring vibration energy; VIF, variance inflation factor.

**Table 4:** Hierarchical multivariable logistic regression models identifying exploratory independent variables associated with right carotid atherosclerosis.

Variables	Model 1			Model 2			Model 3		
	aOR (95% CI)	*p*-Value	VIF	aOR (95% CI)	*p*-Value	VIF	aOR (95% CI)	*p*-Value	VIF
Clinical parameters ^a^									
Advanced age	1.028 (0.225 to 4.692)	0.958	1.20	0.861 (0.177 to 4.181)	0.852	1.27	1.054 (0.160 to 6.954)	0.957	1.33
Male sex	0.271 (0.047 to 1.550)	0.142	1.19	0.296 (0.046 to 1.919)	0.192	1.21	0.307 (0.039 to 2.439)	0.264	1.24
Overweight/obesity	1.678 (0.150 to 18.754)	0.687	1.16	1.275 (0.101 to 16.021)	0.847	1.21	1.350 (0.086 to 21.176)	0.831	1.21
Tobacco use	1.113 (0.240 to 5.168)	0.895	1.22	0.991 (0.182 to 5.408)	0.892	1.47	0.672 (0.089 to 5.109)	0.701	1.50
Hypertension	0.898 (0.158 to 5.097)	0.895	1.26	0.881 (0.141 to 5.514)	0.892	1.32	0.331 (0.030 to 3.693)	0.369	1.40
Hyperlipidemia	1.588 (0.385 to 6.546)	0.531	1.13	2.070 (0.406 to 10.552)	0.360	1.28	1.696 (0.244 to 11.802)	0.593	1.31
SOS, score	0.982 (0.925 to 1.043)	0.534	1.07	0.979 (0.915 to 1.048)	0.537	1.16	0.975 (0.906 to 1.049)	0.493	1.17
Polysomnographic parameters ^b^									
AHI, events/h	–	–	–	1.012 (0.980 to 1.045)	0.445	2.43	1.017 (0.980 to 1.055)	0.369	2.56
Mean SpO_2_, %	–	–	–	0.887 (0.601 to 1.311)	0.524	2.74	0.870 (0.558 to 1.354)	0.537	2.84
Minimum SpO_2_, %	–	–	–	1.134 (0.979 to 1.314)	0.093	3.05	1.156 (0.991 to 1.348)	0.065	3.07
Snoring characteristics									
SSE%-404–500 Hz, %	–	–	–	–	–	–	1.828 (1.160 to 2.882)	** 0.009 **	1.19
SVE%-112–144 Hz, %	–	–	–	–	–	–	1.192 (0.796 to 1.785)	0.394	1.26
Model summary									
*p*-value for Hosmer-Lemeshow test	0.693			0.348			0.396		
Model *χ*^2^	3.922			7.546			21.383		
*p*-value for Omnibus test	0.789			0.673			** 0.044 **		
Nagelkerke *R*^2^	0.103			0.192			0.433		

^a^ Epworth Sleepiness Scale score, apnea index, and 3% oxygen desaturation index were removed to neutralize multicollinearity risks (VIF ≥ 5). ^b^ Bold *p*-values indicate statistical significance (*p* < 0.05). Abbreviations: aOR, adjusted odds ratio; CI, confidence interval; SOS, Snore Outcomes Survey; SSE%, normalized frequency-domain snoring sound energy; SVE%, normalized snoring vibration energy; VIF, variance inflation factor.

## Data Availability

The data presented in this study are available on request from the corresponding author due to ethical reasons.

## Abbreviations

The following abbreviations are used in this manuscript:

ANOVA	one-way analysis of variance
AHI	Apnea–hypopnea index
AI	Artificial intelligence
AUC	Area under the curve
BMI	Body mass index
CCA	Common carotid artery
CI	Confidence interval
CIMT	Carotid intima-media thickness
CVD	Cardiovascular disease
DBP	Diastolic Blood Pressure
DWT	Discrete wavelet transform
ESS	Epworth Sleepiness Scale
FFT	Fast Fourier transform
IQR	Interquartile ranges
LTSA	Long-term spectrum average
NPS	Neck-surface piezoelectric sensor
ODI3	3% oxygen desaturation index
OR	Odds ratio
OSAS	Obstructive sleep apnea syndrome
SAW	Surface acoustic wave
ROC	Receiver operating characteristic
SBP	Systolic blood pressure
SD	Standard deviation
SDB	Sleep-disordered breathing
SOS	Snore Outcomes Survey
SpO_2_	Peripheral oxygen saturation
SSE	Snoring sound energy
SSE%	Normalized snoring sound energy
SVE	Snoring vibratory energy
SVE%	Normalized snoring vibratory energy
VIF	Variance inflation factor
